# Conducting Behavioural Experiments Using an App-Based Self-Help Program for Social Anxiety Disorder (SMASH): Outcomes of a Quasi-Experimental Pre-Post Pilot Trial

**DOI:** 10.32872/cpe.15687

**Published:** 2026-02-27

**Authors:** Johanna S. Schüller, Jacob Kujat, Jan M. Schittenhelm, Ronja von Rechenberg, Antonia Čerič, Jürgen Hoyer, Ulrich Stangier

**Affiliations:** 1 Department for Clinical Psychology and Psychotherapy, Goethe University Frankfurt, Frankfurt, Germany; 2 Mindable Health GmbH, Berlin, Germany; 3 Institute for Clinical Psychology and Psychotherapy, Technische Universität Dresden, Dresden, Germany; Friedrich-Alexander-Universität Erlangen-Nürnberg, Erlangen, Germany

**Keywords:** social anxiety disorder, cognitive behavioural therapy, behavioural experiment, iCBT, app-based, self-help, mental health

## Abstract

**Background:**

Social Anxiety Disorder (SAD) is a prevalent mental disorder characterised by fear of negative evaluation. Although effective treatment approaches are available, access remains limited due to psychological and organisational barriers. Internet-based cognitive behavioural therapy (iCBT) has shown promising results and may facilitate an easy and more resource-efficient access to treatment.

**Method:**

We developed an app-based self-help intervention for SAD based on the Clark and Wells treatment program, implemented as an unguided smartphone application, which was evaluated in this quasi-experimental pre-post pilot study consisting of *N* = 33 patients with a primary diagnosis of SAD. Feasibility was assessed through usage parameters and qualitative feedback. Effectiveness was evaluated in regard to SAD and depression, using clinician-rated measures (LSAS, QIDS-C) at post-treatment (12 weeks) and self-report measures (SPIN, SCQ, BDI-FS) at midpoint and post-treatment. Additionally, moderating effects of usage parameters on symptom reduction were examined.

**Results:**

Clinician- and self-reported SAD symptoms were significantly reduced at post-measurement (within-group effect sizes LSAS: η^2^ = .54; SPIN: η^2^ = .47), with 52% of patients achieving a clinically significant improvement. Despite moderate overall adherence, the amount of conducted behavioural experiments moderated reduction in self-reported SAD symptom severity and SAD-related cognitions. Open feedback supported feasibility and acceptability of the app.

**Conclusion:**

In conclusion, findings provide preliminary support for feasibility, acceptability, and potential effectiveness of *Mindable: Soziale Phobie*. A randomised controlled trial will further evaluate the effectiveness and explore the impact of therapist guidance.

## Highlights

- Newly developed unguided iCBT for SAD demonstrates large pre-post treatment effects on self- and clinician-reported SAD and depressive symptom severity.
- Over 50% of participants experienced reliable improvement in SAD symptoms and anxiety-related beliefs.
- High variance in adherence, with the amount of completed exposures moderating treatment success.

## Background

### Social Anxiety Disorder

Social Anxiety Disorder (SAD) includes fear about negative evaluation in social or performance situations (APA, 2013). SAD symptoms cause considerable suffering, functional impairment and reduced quality of life (Aderka et al., 2012; Lochner et al., 2003). SAD has a lifetime prevalence of 10.7% (Kessler et al., 2012) and is associated with various psychiatric and somatic comorbidities and substantial healthcare costs (Dams et al., 2017; Stein et al., 2017). The Clark and Wells (1995) model explains the psychopathology of SAD by highlighting cognitive and behavioural processes like self-focused attention, negative self-imagery, rumination and safety behaviours. Well-established treatments are available, with cognitive behavioural therapy (CBT) based on the Clark and Wells model (CT-SAD) demonstrating the greatest effectiveness (Mayo-Wilson et al., 2014), including in uncontrolled routine clinical practice (Hoyer et al., 2017). Behavioural experiments (BEs) are the key mechanism driving symptom change (Yilmaz et al., 2025). However, less than 20% of SAD patients receive adequate treatment (Alonso et al., 2018; Stein et al., 2017) due to shame, fear of stigma, uncertainty about treatment access or long waiting times (Goetter et al., 2020; Heinig et al., 2021). Providing CT-SAD requires specific knowledge, competence and time for conducting in-vivo BEs (Clark et al., 2023; Pittig et al., 2019), which further limits availability of treatment.

### Internet-Based CBT

Internet-based CBT (iCBT), delivered via websites or mobile apps, addresses several limitations of face-to-face therapy by increasing accessibility, resource-effectiveness and scalability. Guided iCBT includes therapist support, e.g. in blended formats combining online components with face-to-face sessions, whereas unguided programs are fully self-directed (Käll et al., 2024), and are increasingly provided through mobile apps. Using an iCBT can be less shame-inducing for SAD patients (Goetter et al., 2020) and is explicitly preferred by some patients (Kählke et al., 2019).

iCBTs, including app-based programs, have faced criticism regarding insufficient data security, quality assurance or integration into the healthcare system (e.g. Davies et al., 2020; Denecke et al., 2022; Iwaya et al., 2023). The German DiGA (*Digitale Gesundheitsanwendungen*) program addresses these challenges by providing digital applications that meet strict quality and data security standards, are prescribed by healthcare professionals and reimbursed by health insurances. As part of the Digital Healthcare Act (DVG), DiGAs aim to support diagnosis, monitoring and treatment of diseases (Giebel et al., 2024).

iCBTs have been consistently found to be effective for SAD, with meta-analyses reporting large effects compared to waitlist conditions, including both guided and unguided formats (*g* = 0.76, Pauley et al., 2023; *g* = 0.79, Guo et al., 2021; *g* = 1.31 – 1.34, Andersson et al., 2018). Unguided programs for SAD report a wider range of within-group effect sizes from small (*d* = 0.38, Titov et al., 2008) and moderate (*d* = 0.72, McCall et al., 2018) to large (*d* = 1.17, Kählke et al., 2019).

App-based interventions offer several advantages over web-based iCBTs, including constant availability, which supports the application of strategies in real-world contexts and the conduction of brief, frequent sessions, thereby supporting learning and engagement, and increasing the validity of self-assessments by reducing the reliance on retrospective reporting (Boettcher et al., 2018; Linardon et al., 2024; Stolz et al., 2018). First trials suggest that guided app-based interventions for SAD may be non-inferior to web-based interventions (Stolz et al., 2018) and that combining both approaches can enhance effectiveness (Boettcher et al., 2018). A recent meta-analysis found app-based interventions to reduce SAD symptoms (*g* = .52, Linardon et al., 2024), without differentiating guided from unguided apps. The only trial on an unguided app-based intervention for SAD reports promising results for a brief, unguided intervention focusing on an imaginal exposure (Schwob & Newman, 2023). To date, no other trials have fully evaluated unguided app-based interventions for SAD, underlining the need to investigate their feasibility and clinical efficacy.

The cost-effectiveness potential has been demonstrated for guided iCBTs (Kählke et al., 2022), though few comparisons to active controls are available. Although unguided programs may offer additional cost savings by reducing personnel involvement, mixed evidence on the role of therapist guidance in improving adherence and reducing attrition (Chen et al., 2020; Dryman et al., 2017) raises concerns that a reduction in treatment effectiveness due to lower adherence and retention could offset these savings.

### Research Objectives

In this article, we report findings of a pilot study evaluating the newly developed app-based self-help program *Mindable: Soziale Phobie* for SAD. In line with the Clark and Wells model, in-vivo BEs are the key component. We aimed to investigate three main objectives:

Firstly, we investigated the effectiveness of *Mindable: Soziale Phobie* as stand-alone treatment without therapeutic guidance. We hypothesised that participants would show significant improvements across all outcome measures from pre- to post-treatment. Additionally, we explored clinical relevance of these effects by examining response and remission rates.

Secondly, we examined treatment acceptance, operationalised through treatment expectancy, attrition rates, feedback and adherence measured by app usage parameters. We hypothesised that higher treatment expectancy would be associated with greater adherence.

Lastly, we analysed whether adherence moderated treatment outcomes. Specifically, we hypothesised that the number of conducted *BEs* would moderate treatment outcomes, based on prior findings that greater engagement in behavioural challenges and imaginal exposure exercises is associated with improved outcomes in individuals with SAD (Boettcher et al., 2018; Dryman et al., 2017; Schwob & Newman, 2023).

## Method

### Study Procedures

This pilot study (ethics approval no.: 2022-77) precedes a randomised controlled trial (RCT; ClinicalTrials.gov registration: NCT05554718) to test effectiveness, feasibility and acceptance of the app-based self-help program *Mindable: Soziale Phobie*. Results of this pilot study were used in the authorisation process of the app as digital health application within the German DiGA program (for reference, see Giebel et al., 2024). The study employed a quasi-experimental pre-post design in a naturalistic setting. Recruitment took place from September 2022 to February 2023 through two pathways: therapist referrals from outpatient centers at Goethe University Frankfurt and Technische Universität Dresden, and online recruitment, where interested respondents completed a pre-screening procedure assessing whether self-rated SAD symptom severity (SPIN) met the cut-off of 25 points. Eligible individuals from both pathways were contacted for an on-site intake screening conducted by independent, trained psychologists (at least master’s level), to assess inclusion and exclusion criteria. Inclusion required ages 18 to 65 years, a primary SAD diagnosis as determined by the Structured Clinical Interview for DSM-5 (SCID-5-CV), and absence of more severe or impairing psychiatric disorder. Exclusion criteria included acute suicidal ideation and current psychotherapeutic or psychopharmacological treatment. See Supplementary Figure 1 for the participant flowchart. Clinician-rated outcomes were assessed at baseline- and post-measurement (Week 12), self-reported questionnaires additionally at midpoint (Week 6). After enrollment, participants received a download link and access code, which was linked to the individual via a linking log stored separately from identifying data. The app was accessible on smartphones or tablets running iOS 12/Android 7 or higher, with a stable internet connection. For more details on procedure, see the RCT study protocol (Schittenhelm et al., 2023).

### Participants

Eligible patients after pre-screening or based on referral (*N* = 39) were screened for intake. Of these, 33 were included in the pilot study. 76% of participants were female and mean age was *M* = 31.97, *SD* = 9.14 (Range 19 – 65). Sociodemographic data is shown in Supplementary Table 2.

### Intervention

*Mindable: Soziale Phobie* translates the German adaptation (Stangier et al., 2016) of the CT-SAD treatment (Clark & Wells, 1995) into a smartphone program. In this study, the program was implemented as a fully unguided self-help program; however, it can also be used to bridge waiting times for psychotherapy or in a blended treatment alongside face-to-face therapy.

The program consists of three *structured* and two *unstructured modules*. *Structured modules*, completed in a fixed sequence, include *psychoeducation*, *development of an individualised case formulation*, and a *video-based processing experiment* illustrating the effects of self-focused attention and safety behaviours. These could easily be completed within one week, though users proceed at their own pace. Afterwards, users access the *unstructured modules*, comprising *attention training (AT)* and *behavioural experiments (BEs)*. *BEs* form a core therapeutic component, aimed at testing maladaptive beliefs through real-life exposure tasks without safety behaviours. The app supports users in planning and reflection, but provides no reminders. *BEs* can be undertaken flexibly, with participants encouraged to complete as many as possible during the 12-week program.

### Outcome Measures

#### Attrition, Adherence, and Participant Feedback

Attrition was defined as the proportion of patients who did not complete the midpoint or post measure. Adherence was defined as the extent to which participants engaged with the intervention as intended and was operationalised by tracking the number of conducted *psychoeducation modules*, *behavioural experiments (BE)*, *attention trainings (AT),* and *usage days*. Participants rated their expectancy of iCBT effectiveness for treating mental disorders on a scale from 0 to 100. At post-treatment, feedback was collected through open-ended questions.

#### Clinical Diagnoses

SAD diagnoses were obtained using the SCID-5-CV (First et al., 2016; German version: Beesdo-Baum et al., 2019a) at the intake screening. We used a short screening scale for Borderline Personality Disorder (Short-Bord; Wongpakaran et al., 2019) and conducted relevant sections of the SCID-5-PD (First et al., 2015; German version: Beesdo-Baum et al., 2019b) if there were indicators of the presence of a personality disorder.

#### Self- and Clinician-Reported Measures

All questionnaires used are well-established measures with solid psychometric properties. See Supplementary Table 1 for psychometric data. We report results for main outcomes here and present results for additional self-rated measures in Supplementary Tables 3 to 5. Main outcomes included clinician-rated (Liebowitz Social Anxiety Scale; LSAS) and self-rated (Social Phobia Inventory; SPIN) SAD symptom severity as well as clinician-rated (Quick Inventory of Depressive Symptoms, Clinician-Rated Version; QIDS-C) and self-rated (Beck Depression Inventory - Fast Screen; BDI-FS) depressive symptom severity. In addition, anxiety-related beliefs were assessed using the Social Cognitions Questionnaire (SCQ) with subscales for frequency and intensity of beliefs.

### Data Analysis

The sample size for this pilot study was determined through simulation-based power analysis using “simr” (Green & MacLeod, 2016). Based on a simplified linear mixed model (LMM) corresponding to a one-group pre-post design of the APP condition described in the study protocol by Schittenhelm et al. (2023), we iteratively increased the number of simulated cases until the estimated power exceeded 80%. Analyses were performed with the intention-to-treat (ITT) sample, consisting of all participants included. Missing data was imputed using a non-parametric Random Forest algorithm (Stekhoven & Bühlmann, 2012). Linear mixed models (LMMs) were used to determine change between measurements. We calculated partial η^2^ as effect size, defining a small effect as η^2^ > .01, medium effect as η^2^ > .06 and η^2^ > .14 as large effect (Cohen, 1988). Treatment response was defined as LSAS sum score reduction of at least 29% and remission as a LSAS sum score below 30 (von Glischinski et al., 2018). Reliable clinical change indices (RCI) were determined following Jacobson and Truax (1991). Moderation analyses were conducted using regression models including interaction terms between time and usage parameters, with baseline scores included as covariates. All analyses were performed using R Statistical Software (v4.2.2; R Core Team, 2021). The reporting of this pilot study was guided by the CONSORT-EHEALTH guidelines for digital health interventions (Eysenbach et al., 2011), with adaptations due to the scope and format of a short report.

## Results

### Improvement of Symptomatology

Descriptively, all outcomes improved over the study period, with self-rated measures improving by midpoint and remaining stable thereafter. Means and standard deviations for all measures and time points are provided in Supplementary Table 3. LMMs were used to evaluate statistical significance of changes from baseline- to midpoint- and post-measurement (Table 1). LSAS significantly improved from baseline to post-measurement, while QIDS-C did not. SPIN and BDI-FS improved by study midpoint and remained stable afterwards. See Supplementary Table 4 and Supplementary Figure 2 for detailed results including additional measures.

**Table 1:** Linear Mixed Model Results for all Outcomes, Intention-To-Treat Data Set (N = 33)

Variable	*F* ^a^	*p* ^a^	Within-group $\eta_{p}^{2}$ [95% CI]	Midpoint (6 weeks)			Post (12 weeks)		
				β^b^	*S* ^b^	*p* ^b^	β^b^	*SE* ^b^	*p* ^b^
LSAS Total	38.18	*	.54 [.30, .70]	/	/	/	-21.55	3.49	*
SPIN	28.93	*	.47 [.29, .60]	-10.91	1.74	*	-11.97	1.74	*
SCQ Frequency	28.93	*	.37 [.18, .52]	-0.46	0.10	*	-0.54	0.10	*
SCQ Conviction	18.89	*	.31 [.12, .46]	-8.87	2.51	*	-13.05	2.51	*
QIDS-C	14.07	.539	.01 [.00, .17]	/	/	/	-0.42	0.68	.539
BDI-FS	11.98	*	.27 [.10, .43]	-4.55	1.12	*	-4.91	1.12	*

*Note.* LSAS = Liebowitz Social Anxiety Scale; SPIN = Social Phobia Inventory; SCQ = Social Cognitions Questionnaire; QIDS-C = Quick Inventory of Depressive Symptoms; BDI-FS = Beck Depression Inventory. See Supplementary Table 4 for results on additional measures.

^a^*F*- and *p*-values derived from *F*-tests of linear mixed models. ^b^β-values with standard errors and according *p*-values derived from coefficients of linear mixed models using Satterthwaite's method.

*p < .001.

### Response and Remission

Treatment response was achieved by 17 participants (52%). Only one patient reached remission. Reliable clinical change in SAD symptom severity was observed in 67% of participants based on clinician ratings (LSAS) and in 61% based on self-ratings (SPIN) at post-measurement. Notably, all self-rated improvements were achieved by midpoint. For an overview of RCI outcomes, see Supplementary Table 5.

### Attrition, Adherence and Feedback

22 participants (67%) completed all study procedures, eleven participants (33%) dropped out either before (*N* = 4) or after (*N* = 7) midpoint-measurement. The mean duration of study participation among drop-outs was *M* = 41.82 days, *SD* = 15.65. Reasons for drop-out included initiating psychotherapeutic or psychopharmacologic treatment (*N* = 3) and dissatisfaction with the intervention (*N* = 2). For six participants, the drop-out reasons were unknown. On average, patients used the app on *M* = 8.18 days, *SD* = 6.78, spread out over a mean duration of *M* = 49.45 days, *SD* = 31.47. Most participants completed all three *psychoeducation modules* (97%) and all three *model creation modules* (70%), which were both part of the *structured modules*. 39% of participants conducted at least one *BE*, with a maximum of 13 experiments. The number of usage days was correlated more strongly with the number of *unstructured modules* (*BE: r_s_* = .85, *p* < .001; *AT: r_s_* = .83, *p* < .001) than *structured modules* (*r_s_* = .49, *p* = .004). A priori treatment expectancy was moderate with *M* = 68.52, *SD* = 19.41, and was not correlated with any usage parameter. See Supplementary Table 6 for descriptive data on usage parameters. An overview of open feedback is presented in Supplementary Table 7.

### Moderation Analyses

The number of conducted *BEs* moderated SAD symptom severity (SPIN) and both intensity and frequency of anxiety-related cognitions (SPQ), but not clinician-rated symptom severity (LSAS). Specifically, a higher number of BEs was associated with greater reductions in SAD symptoms and cognitions. A similar effect was observed for AT: both the number of *ATs* and the number of *usage days* moderated self-rated SAD symptom severity (SPIN) and the frequency of anxiety-related cognitions (SPQ), with greater engagement linked to stronger symptom improvement. The number of completed *structured modules* did not moderate any outcome. See Table 2 for detailed results.

**Table 2:** Outcomes of Moderation Analyses

Variable	*df*	Structured Modules		Behavioural Experiments		Attention Trainings		Usage Days	
		*t*	*p*	*t*	*p*	*t*	*p*	*t*	*p*
LSAS	31	0.44	.67	-0.47	.64	-0.56	.58	-0.17	.86
SPIN	62	-1.33	.19	-2.52	**.01**	-2.20	**.03**	-2.13	**.04**
SCQ Frequency	62	-0.39	.70	-2.25	**.03**	-1.77	.08	-1.54	.13
SCQ Conviction	62	-1.50	.14	-2.25	**.03**	-2.10	**.04**	-2.05	**.04**

*Note.* Results of moderation analyses showing the moderating effect of each usage parameter (listed in the title row) on each outcome. Boldface *p* values denote statistical significance at α = .05.

## Discussion

### Treatment Effectiveness

The app-based self-help program *Mindable: Soziale Phobie* demonstrated a very large pre-post treatment effect on clinician-rated (LSAS: η^2^ = .54) and self-reported SAD symptoms (SPIN: η^2^ = .47. These effects are in line with, and in part exceed, those reported in previous studies on unguided web-based iCBT programs for SAD (e.g. Kählke et al., 2019; McCall et al., 2018; Titov et al., 2008). Large effects were also observed in anxiety-related cognitions and depressive symptoms. Over half of the participants experienced reliable clinical improvement in SAD symptom severity and beliefs. Consistent with prior evidence, our study’s pre-post treatment effects are in the same range as face-to-face CBT (*d* = 1.22, Bandelow et al., 2015). Notably, these effects were achieved without clinician guidance, highlighting the potential of app-based self-help for SAD.

### Attrition and Treatment Adherence

Despite large treatment effects, attrition and adherence remain challenges in unguided app interventions. Our program’s drop-out rate of 33% was higher than in face-to-face CBT for SAD (e.g. Stangier et al., 2011; Clark et al., 2006), but lower than most unguided internet-based CBT trials, which report rates up to 66% (Chen et al., 2020; McCall et al., 2018; Titov et al., 2008). Our comparatively low drop-out may be attributed to the app-based format, which offers greater flexibility and easier integration into daily life.

Adherence declined over time, with most patients completing the initial *structured modules*, but 61% of participants not conducting any *BEs*. This aligns with Dryman et al. (2017), who report that one third of users dropped out after cognitive restructuring but before completing exposures, potentially due to avoidance or task difficulty. Alternatively, users might have disengaged after an initial symptom reduction, as reflected in our finding that improvements occurred mostly in the first half of the study. Notably, patients who engaged more in *unstructured modules*, specifically *BEs*, showed greater reductions in SAD symptoms and beliefs, consistent with findings identifying BEs as a key mechanism in CT-SAD (Yilmaz et al., 2025).

Results on attrition and adherence raise questions about the impact of lacking therapist guidance. While some participants independently engaged fully, others who dropped out early might have benefited from additional support, as suggested in participant feedback indicating a desire for external encouragement (see Supplementary Table 2). Other participants, however, valued the flexibility and anonymity of the self-guided format, consistent with previous observations (Kählke et al., 2019). These findings highlight individual differences in treatment needs, emphasising the importance of future research to identify differentiating patient characteristics in order to optimise adherence, effectiveness, and resource allocation in digital interventions (Ibeh et al., 2024).

### Limitations

First, as this was a pilot study assessing the feasibility of an app-based self-help intervention, the small sample size limits external validity. Second, the uncontrolled design prevents firm conclusions about efficacy, so results should be interpreted with caution. Third, selective sampling may restrict the generalizability to the population of SAD patients. For example, digital affinity may be higher among younger individuals, while older patients may prefer face-to-face interaction. This could lead to an overestimation of efficacy in the general population. Fourth, the 12-week study duration limits assessment of long-term engagement or sustained effects and short-term improvements observed may not persist over time. Fifth, participants were not blinded due to the nature of the intervention, which may further contribute to expectancy effects or reporting bias. Finally, the app was tested as a standalone tool, whereas in real-world clinical settings such tools are more commonly used as part of therapist-guided treatments.

### Future Directions

Future research should investigate the effectiveness of app-based self-help interventions in an RCT with a larger and more diverse patient sample. A key area for further study is the specific contribution of app usage to the efficacy of therapist-guided treatments (Schittenhelm et al., 2023; Stolz et al., 2018), and their benefit for subgroups of patients less inclined to blended care, such as older adults. Several of these questions are currently being explored in an ongoing RCT (Schittenhelm et al., 2023), which compares unguided app use, guided use with additional therapy sessions, and a waitlist control condition. This study also examines predictors of treatment success, including patient characteristics and mechanisms of change, monitored through weekly symptom assessments.

### Conclusion

iCBTs offer a quickly available, effective, and potentially resource-efficient treatment option for highly self-motivated patients or patients who feel too ashamed to pursue face-to-face psychotherapy. This pilot study provides initial evidence for the effectiveness of an app-based self-help program implementing CT-SAD based on the Clark and Wells model. Despite moderate overall treatment engagement, the results demonstrate significant improvements in SAD symptomatology. Further research on its implementation and the potential benefit of therapist guidance is warranted.

## Supplementary Materials

The Supplementary Materials contain the following items:

- Preregistration (Schüller et al., 2022S)
- Supplementary figures and tables (Schüller et al., 2026S)
  - *Supplementary Figure 1:* Study flow chart
  - *Supplementary Figure 2:* Results for patient- and clinician-rated outcomes
  - *Supplementary Table 1:* Overview of clinician-rated and self-report questionnaires used in the study
  - *Supplementary Table 2:* Sociodemographic data of study participants
  - *Supplementary Table 3:* Descriptive data of all outcomes for baseline-, midpoint- (6 weeks) and post-measurement (12 weeks), intention-to-treat data set
  - *Supplementary Table 4:* Linear mixed model results for all outcomes, intention-to-treat data set
  - *Supplementary Table 5:* Reliable change indices and response rates for study outcomes, intention-to-treat data set
  - *Supplementary Table 6:* Descriptive data for usage parameters
  - *Supplementary Table 7:* Overview of the responses to the open-format feedback survey

SchüllerJ. S.
KujatJ.
SchittenhelmJ. M.
von RechenbergR.
ČeričA.
HoyerJ.
StangierU.
 (2022S). Evaluation of a smartphone application for self-help for social anxiety (SMASH)
[Preregistration; Registration No.: NCT05554718]. PsychOpen. https://clinicaltrials.gov/study/NCT05554718
10.1186/s13063-023-07168-5PMC997439236855058

SchüllerJ. S.
KujatJ.
SchittenhelmJ. M.
von RechenbergR.
ČeričA.
HoyerJ.
StangierU.
 (2026S). Supplementary materials to "Conducting behavioural experiments using an app-based self-help program for social anxiety disorder (SMASH): Outcomes of a quasi-experimental pre-post pilot trial"
[Supplementary figures and tables]. PsychOpen. 10.23668/psycharchives.21616
PMC1295530341783467

## Data Availability

As the data collected is particularly sensitive personal health data, it can be accessed in pseudonymised form and only in the case of legitimate interest upon request from the Department for Clinical Psychology and Psychotherapy, Goethe University Frankfurt, Frankfurt, Germany. R scripts and study materials used here can also be accessed on request by contacting the same institute.

## Preregistration

The entire research project (the RCT and the associated pilot study presented here) was preregistered on ClinicalTrials.gov (registration number: NCT05554718; see Schüller et al., 2022S).

## Reporting Guidelines

The reporting of this pilot study was guided by the CONSORT-EHEALTH guidelines for digital health interventions, with adaptations due to the scope and format of a short report.

## Author Note

The authors made the following contributions: *Johanna S. Schüller*: Intervention Development, Recruitment, Trial Management, Data Collection, Formal Analysis, Writing - Original Draft Preparation, Writing - Review & Editing; *Jacob Kujat*: Conceptualization, Intervention Development, Trial Management, Formal Analysis, Writing - Original Draft Preparation, Writing - Review & Editing; *Jan M. Schittenhelm*: Intervention Development, Recruitment, Trial Management, Data Collection, Formal Analysis, Writing - Original Draft Preparation, Writing - Review & Editing; *Ronja von Rechenberg*: Intervention Development, Recruitment, Trial Management, Data Collection, Formal Analysis, Writing - Review & Editing; *Antonia Čerič*: Recruitment, Trial Management, Data Collection, Writing - Review & Editing; *Jürgen Hoyer*: Conceptualization, Intervention Development, Trial Management, Review & Editing, Supervision; *Ulrich Stangier*: Conceptualization, Intervention Development, Trial Management, Review & Editing, Supervision.
